# Additional adjuvant radiotherapy improves survival at 1 year after surgical treatment for pancreatic cancer patients with T_4_, N_2_ disease, positive resection margin, and receiving adjuvant chemotherapy

**DOI:** 10.3389/fonc.2023.1109068

**Published:** 2023-07-18

**Authors:** Lili Wu, Yaolin Xu, Yuhong Zhou, Zhaochong Zeng, Yue Fan, Dansong Wang, Wenchuan Wu, Xi Guo, Minzhi Lv, Yuxiu Ouyang, Shisuo Du, Wenhui Lou

**Affiliations:** ^1^ Department of Radiotherapy, Zhongshan Hospital Fudan University, Shanghai, China; ^2^ Department of Pancreatic Surgery, Zhongshan Hospital Fudan University, Shanghai, China; ^3^ Cancer Center, Zhongshan Hospital Fudan University, Shanghai, China; ^4^ Department of General Surgery, Zhongshan Hospital Fudan University, Shanghai, China; ^5^ Department of Medical Oncology, Zhongshan Hospital Fudan University, Shanghai, China; ^6^ Department of Traditional Chinese Medicine, Zhongshan Hospital Fudan University, Shanghai, China; ^7^ Department of Biostatistics, Zhongshan Hospital Fudan University, Shanghai, China; ^8^ Department of Abdominal Tumor Radiotherapy, Guangdong Province Zhongshan City People's Hospital, Zhongshan, Guangdong, China

**Keywords:** pancreatic ductal adenocarcinoma, radiotherapy, chemotherapy, surgery, overall survival

## Abstract

**Background:**

While adjuvant chemotherapy has been established as standard practice following radical resection of pancreatic ductal adenocarcinoma (PDAC), the role of adjuvant radiation therapy (RT) and which patients may benefit remains unclear.

**Methods:**

This retrospective study included PDAC patients who received pancreatic surgery from April 2012 to December 2019 in Zhongshan Hospital Fudan University. Patients with carcinoma *in situ*, distant metastasis, and without adjuvant chemotherapy were excluded. Cox proportional hazards modeling of survival were constructed to find potential prognostic factors. Propensity score matching (PSM) and exploratory subgroup analyses were used to create a balanced covariate distribution between groups and to investigate therapeutic effect of radiotherapy in certain subgroups.

**Results:**

A total of 399 patients were finally included, 93 of them receiving adjuvant chemoradiotherapy (C+R+) and 306 of them receiving chemotherapy only. Patients in C+R+ group were more likely to be male patients with T3-4 disease. Lymph node metastases was the only negative prognostic factor associated with overall survival (OS). Additional adjuvant RT was not associated with an OS benefit both before and after PSM. Surprisingly, a trend towards improved OS with RT among patients with either T4, N2 disease or R1 resection becomes significant in patients alive more than 1 year after surgery.

**Conclusion:**

Adjuvant RT was not associated with an OS benefit across all patients, though did show a possible OS benefit for the subgroup with T4N2 disease or R1 resection at 1 year after surgery.

## 1 Introduction

Pancreatic ductal adenocarcinoma (PDAC) is one of the malignancies with the poorest prognosis, with an estimated 5-year survival around 10% (1). In China, the estimated numbers of new cases and deaths per year were 125,000 and 122,000, respectively (2, 3). Nowadays, PDAC has been recognized as a systemic disease and multimodality management is highly recommended in Chinese Society of Clinical Oncology (CSCO), National Comprehensive Cancer Network (NCCN), and European Society for Medical Oncology (ESMO) practice guidelines for pancreatic cancer (4–7).

Adjuvant chemotherapy has been standard-of-care for PDAC after curative surgery. Modified fluorouracil (5-FU), leucovorin, irinotecan, and oxaliplatin (mFOLFIRINOX) and nab-paclitaxel plus gemcitabine have been shown to significantly improve survival outcomes of PDAC patients after surgery (8–10). For those who could not tolerate the side effects of combined regimens, S-1 or gemcitabine (GEM) alone was alternative treatment and could also prolonged overall survival after surgical resection (11). However, whether additional adjuvant radiotherapy would bring survival benefit remained controversial.

Previous Gastrointestinal Tumor Study Group (GITSG) 9173 trial and some retrospective studies had demonstrated that chemoradiotherapy (CRT) showed relatively good local control and brought survival benefit for PDAC patients after surgical treatment compared with those without any adjuvant treatment (12–15). Meanwhile, the Radiation Therapy Oncology Group (RTOG) 0848, 9704 trials, and two other prospective studies had all indicated promising efficacy of either 5-FU or GEM based CRT in adjuvant setting among PDAC patients (16–21).

When directly comparing adjuvant CRT with chemotherapy alone, the results of EORTC-40013-22012/FFCD-9203/GERCOR and ESPAC-1 trials were contradictory. Karin et al. administrated either GEM based CRT or GEM to pancreatic head cancer patients after R0 resection of primary tumor. Median disease-free survival (DFS) and overall survival (OS) showed no difference between groups, while first local recurrence was less frequent in CRT group (22). However, the results of ESPAC-1 indicated that CRT group (including CRT alone and CRT + chemotherapy) was associated with poorer OS comparing with no CRT group (including observation and chemotherapy alone) (23, 24).

One of the reasons for these discrepancies may be patient selection for adjuvant CRT. Which specific patient subgroups could benefit from adjuvant CRT remained unknown. We previously reported that S-1 based CRT showed promising efficacy and well-tolerated in terms of adverse effect in resected PDAC patients with high-risk pathologic feature (including positive resection margin, pathologic T3-4 and/or N1-2 disease, peripancreatic fat invasion, microvascular invasion, and perineural invasion) (25). Some other recent retrospective studies with either single center cohort or public database indicated possible benefit of CRT for PDAC patients with T3 disease, lymph node metastases, and R1 resection (15, 26–28). Meanwhile, failure to adhere to radiotherapy protocol among different medical centers may have also had an impact on the survival outcome (29). As a high-volume pancreatic cancer center with experienced radiation therapists, we conducted the present retrospective cohort study to further explore the therapeutic effect of adjuvant CRT in PDAC patients after surgery.

## 2 Materials and methods

### 2.1 Patients

This study was reviewed and approved by the ethics committee of Zhongshan Hospital Fudan University. We included patients who underwent curative-intent surgery between 1 April 2012 and 31 December 2019 and were pathologically diagnosed as pancreatic ductal adenocarcinoma. R1 resection was defined as a positive margin within less than 1 mm according to the 8th American Joint Committee on Cancer (AJCC) manual. Patients with carcinoma *in situ* (Tis) or distant metastases, and those not receiving adjuvant chemotherapy were excluded. All the patients were restaged pathologically according to the 8th AJCC TNM classification. OS was calculated as the time from surgery to death or last follow-up. DFS was calculated as the time from surgery to disease progression. The follow-up duration was from the surgery date to 1 July 2022. All the medical information and time of survival were obtained from medical records and telephone interviews. Physical examinations, complete blood count (CBC), liver function, kidney function, and CA 19-9 radioimmunoassay were tested before the start of treatment and repeated every cycle of chemotherapy. Contrast-enhanced computed tomography and/or magnetic resonance imaging were performed every other cycle of chemotherapy and 3 weeks after CRT. Evaluations of treatment response according to the radiology reports were done by the same oncologist in a blinded manner. This study was approved by the ethical committee of our hospital (B2022-249R) and all patients have signed informed consent forms before we collected their medical records for researching purpose.

### 2.2 Statistical analysis

Data analyses were performed by R project 4.2.1 for Windows and Rstudio 2022.07.1. Normality and homogeneity of variance were tested by Shapiro-Wilk test and Levene’s test. Categorical variables were reported as frequencies and percentages. Continuous variables conforming to normal distribution were presented by means and standard error, others were described as medians and Inter-Quartile Range. The baseline characteristics between different groups were compared using Fisher’s precision probability test for categorical variables, using Wilcoxon rank sum test for continuous variables, respectively. Propensity score matching (PSM) was performed with “MatchIt” packages using R project. A 1:1 ratio propensity score matching study group was created using the nearest neighbor matching method with a 0.6 caliper. Survival curves were drawn with the method of Kaplan–Meier, and log-rank test and landmark analyses was used to compare the survival outcome of different groups. Cox proportional hazards model was used to estimate the hazard ratio of death. The significant statistical variables (p<0.1) in univariate Cox regression analysis were incorporated into the multivariate analysis to identify the independent prognostic factors for survival. And forest plot was performed to show the outcome of subgroup analysis.

## 3 Results

### 3.1 Baseline characteristics

**Figure 1:**
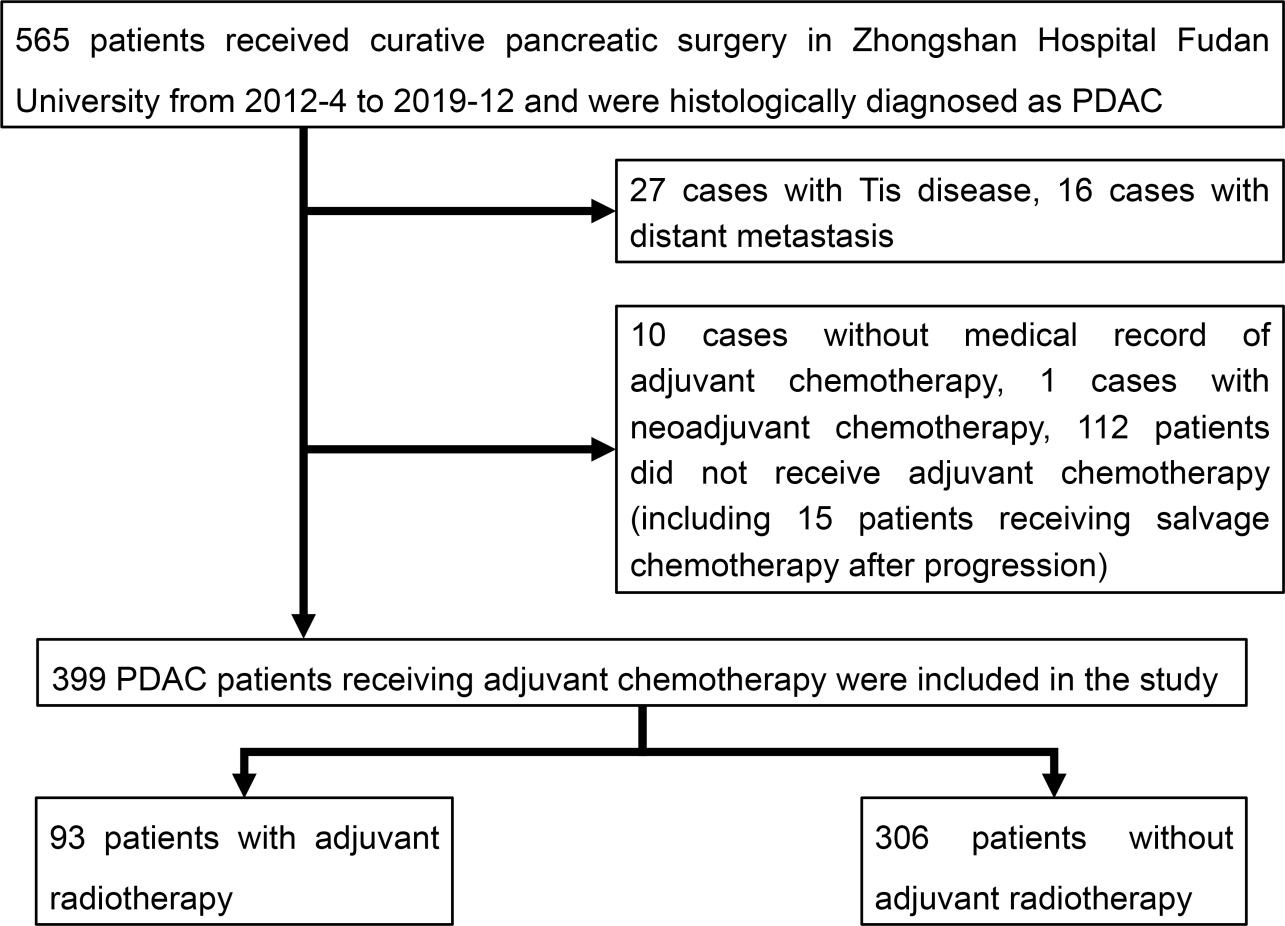
Flowchart of patient selection. PDAC, pancreatic ductal adenocarcinoma.

A total of 399 patients were incorporated in the total cohort and were stratified by whether receiving adjuvant radiotherapy or not. Flowchart of patient selection were illustrated in **Figure 1** and patients’ clinicopathologic characteristics were listed in **Table 1**. There were 306 patients receiving adjuvant chemotherapy only (C+R-) and 93 patients receiving both adjuvant chemotherapy and radiotherapy (C+R+). Patients in C+R+ groups were more likely to be male patients (Male, C+R- vs C+R+, 55.9% vs 72%, p=0.006) with pathologic T3-4 disease (T3-4, C+R- vs C+R+, 15.3% vs 28%, p=0.008). Other factors, including N stage and tumor markers, didn’t differ significantly between two groups.

**Table 1:** Baseline characteristics of patients stratified by adjuvant treatment.

	Total	Number of Patient (n=399)		P-value
		C+R- (n=306)	C+R+ (n=93)	
Age				
Mean (SD)	62.7 ( ± 8.6)	62.9 ( ± 8.8)	61.9 ( ± 7.8)	0.22
Gender				
Male	238 (59.6%)	171 (55.9%)	67 (72.0%)	0.006
Female	161 (40.4%)	135 (44.1%)	26 (28.0%)	
ASA classification				
1	57 (14.3%)	44 (14.4%)	13 (14.0%)	0.75
2	328 (82.2%)	252 (82.4%)	76 (81.7%)	
3	12 (3.0%)	9 (2.9%)	3 (3.2%)	
Unknown	2 (0.5%)	1 (0.3%)	1 (1.1%)	
Primary tumor location on pancreas				
Head	209 (52.4%)	165 (53.9%)	44 (47.3%)	0.51
Body & Tail	183 (45.9%)	135 (44.1%)	48 (51.6%)	
Total Pancreas	7 (1.8%)	6 (2.0%)	1 (1.1%)	
Primary Tumor				
T1	92 (23.1%)	68 (22.2%)	24 (25.8%)	0.008
T2	234 (58.6%)	191 (62.4%)	43 (46.2%)	
T3	62 (15.5%)	38 (12.4%)	24 (25.8%)	
T4	11 (2.8%)	9 (2.9%)	2 (2.2%)	
Regional Lymph Nodes				
N0	226 (56.6%)	177 (57.8%)	49 (52.7%)	0.13
N1	145 (36.3%)	112 (36.6%)	33 (35.5%)	
N2	26 (6.5%)	15 (4.9%)	11 (11.8%)	
Unknown	2 (0.5%)	2 (0.7%)	0 (0.0%)	
AJCC 8th stage				
IA	62 (15.5%)	48 (15.7%)	14 (15.1%)	0.1
IB	129 (32.3%)	107 (35.0%)	22 (23.7%)	
IIA	33 (8.3%)	22 (7.2%)	11 (11.8%)	
IIB	138 (34.6%)	105 (34.3%)	33 (35.5%)	
III	37 (9.3%)	24 (7.8%)	13 (14.0%)	
Microvascular Invasion				
No	332 (83.2%)	252 (82.4%)	80 (86.0%)	0.53
Yes	67 (16.8%)	54 (17.6%)	13 (14.0%)	
Nerve Invasion				
No	67 (16.8%)	55 (18.0%)	12 (12.9%)	0.27
Yes	332 (83.2%)	251 (82.0%)	81 (87.1%)	
Fat Invasion				
No	79 (19.8%)	63 (20.6%)	16 (17.2%)	0.55
Yes	320 (80.2%)	243 (79.4%)	77 (82.8%)	
Resection Margin				
Negative	374 (93.7%)	290 (94.8%)	84 (90.3%)	0.14
Positive	25 (6.3%)	16 (5.2%)	9 (9.7%)	
Tumor Deposits				
No	367 (92.0%)	279 (91.2%)	88 (94.6%)	0.38
Yes	32 (8.0%)	27 (8.8%)	5 (5.4%)	
Tumor Differentiation				
Well differentiated	4 (1.0%)	4 (1.3%)	0 (0.0%)	0.78
Moderately differentiated	168 (42.1%)	126 (41.2%)	42 (45.2%)	
Poorly differentiated	220 (55.1%)	170 (55.6%)	50 (53.8%)	
Unknown	7 (1.8%)	6 (2.0%)	1 (1.1%)	
CA 19-9 (U/ml)				
<35	95 (23.8%)	75 (24.5%)	20 (21.5%)	0.21
35-200	153 (38.3%)	110 (35.9%)	43 (46.2%)	
>200	151 (37.8%)	121 (39.5%)	30 (32.3%)	
CA 125 (U/ml)				
<20	222 (70.3%)	169 (69.5%)	53 (72.6%)	0.66
>=20	94 (29.7%)	74 (30.5%)	20 (27.4%)	
CEA (ng/ml)				
<5	296 (75.1%)	227 (75.2%)	69 (75.0%)	1
>=5	98 (24.9%)	75 (24.8%)	23 (25.0%)	
AFP (ng/ml)				
<20	387 (99.7%)	297 (99.7%)	90 (100.0%)	1
>=20	1 (0.3%)	1 (0.3%)	0 (0.0%)	
CA50 (U/ml)				
<25	67 (24.8%)	50 (24.0%)	17 (27.4%)	0.62
>=25	203 (75.2%)	158 (76.0%)	45 (72.6%)	
CA153 (U/ml)				
<25	193 (94.6%)	141 (94.6%)	52 (94.5%)	1
>=25	11 (5.4%)	8 (5.4%)	3 (5.5%)	
CA242 (U/ml)				
<29	144 (53.1%)	110 (52.6%)	34 (54.8%)	0.77
>=29	127 (46.9%)	99 (47.4%)	28 (45.2%)	
CA724 (U/ml)				
<10	290 (90.3%)	222 (89.5%)	68 (93.2%)	0.5
>=10	31 (9.7%)	26 (10.5%)	5 (6.8%)	
Portal Vein Resection				
No	368 (92.2%)	284 (92.8%)	84 (90.3%)	0.51
Yes	31 (7.8%)	22 (7.2%)	9 (9.7%)	
Artery (SMA, HA, CA, LGA) Resection				
No	386 (96.7%)	295 (96.4%)	91 (97.8%)	0.74
Yes	13 (3.3%)	11 (3.6%)	2 (2.2%)	

SD, standard deviation; ASA, American Society of Anesthesiologists; AJCC, American Joint Committee on Cancer; CA 19-9, carbohydrate antigen 19-9; CA 125, carbohydrate antigen 125; CEA, carcinoembryonic antigen; AFP, alpha-fetoprotein; CA 50, carbohydrate antigen 50; CA 153, carbohydrate antigen 50; CA 242, carbohydrate antigen 242; CA 724, carbohydrate antigen 724; SMA, superior mesenteric artery; HA, hepatic artery; CA, celiac trunk; LGA, left gastric artery.

### 3.2 Survival comparison and potential prognostic factors

**Figure 2:**
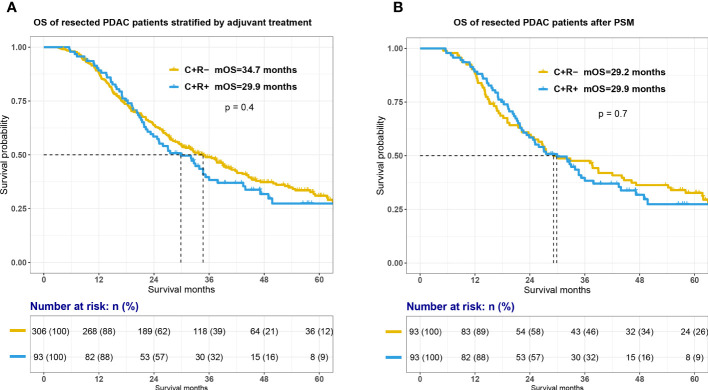
Survival between C+R- and C+R+ groups before and after propensity score matching. Figure showed the survival curves between two groups before **(A)** and after propensity score matching **(B)**. OS, overall survival; C+R-, receiving adjuvant chemotherapy only; C+R+, receiving both adjuvant chemotherapy and radiotherapy; PSM, propensity score matching.

The Kaplan-Meier estimator were used to compare survival outcome between C+R-and C+R+ groups. As shown in **Figure 2A**, the addition of adjuvant radiotherapy to resection and adjuvant chemotherapy was not associated with an increase in OS among all PDAC patients (mOS, C+R- vs C+R+, 34.7 vs 29.9 months, p=0.4). Cox proportional-hazards model were constructed to further investigate potential prognostic factors among PDAC patients with surgical treatment and adjuvant chemotherapy. The univariate and multivariate Cox regression analysis demonstrated that regional lymph node metastases was an independent prognostic factor (p=0.04; N0 as reference; N2, hazard ratio [HR]=2.71, 95% confidential index [CI] 1.21-6.07, p=0.015) and preoperative carbohydrate antigen 125 [CA 125] level seemed to associated with OS (p=0.061; CA 125<20 U/ml as reference; CA 125≥20 U/ml, HR 1.59, 95% CI 0.85-3.8). Other details of Cox proportional model was summarized in **Table 2**.

**Table 2:** Cox proportional analyses of OS in total cohort.

	Univariate		Multivariate	
	HR	P-value	HR	P-value
Age				
Mean (SD)	1.01 (0.9951-1.024)	0.195		
Gender				
Male	1 (As reference)	0.843		
Female	1.03 (0.797-1.32)			
ASA classification				
1	1 (As reference)	0.463		
2	0.769 (0.546-1.08)	0.133		
3	0.697 (0.311-1.56)	0.379		
Primary tumor location on pancreas				
Head	1 (As reference)	<0.01	1 (As reference)	0.283
Body & Tail	0.636 (0.492-0.821)	<0.01	0.737 (0.487-1.115)	0.148
Total Pancreas	0.717 (0.265-1.94)	0.512	0.47 (0.0631-3.51)	0.462
Primary Tumor				
T1	1 (As reference)	0.328		
T2	1.23 (0.898-1.69)	0.196		
T3	1.27 (0.838-1.93)	0.258		
T4	1.83 (0.901-3.73)	0.094		
Regional Lymph Nodes				
N0	1 (As reference)	<0.01	1 (As reference)	0.04
N1	1.51 (1.16-1.97)	<0.01	2.38 (0.67-8.44)	0.18
N2	3.42 (2.2-5.32)	<0.01	2.71 (1.21-6.07)	0.015
AJCC 8th stage				
IA	1 (As reference)	<0.01	1 (As reference)	0.426
IB	1.23 (0.809-1.86)	0.355	1.2 (0.656-2.21)	0.548
IIA	1.07 (0.598-1.91)	0.820	0.575 (0.19-1.74)	0.327
IIB	1.64 (1.09-2.47)	0.017	0.617 (0.183-2.08)	0.436
III	3.2 (1.95-5.23)	<0.01	1.15 (0.267-4.910)	0.855
Microvascular Invasion				
No	1 (As reference)	0.064	1 (As reference)	0.397
Yes	1.36 (0.984-1.87)		1.22 (0.767-1.96)	
Nerve Invasion				
No	1 (As reference)	0.010	1 (As reference)	0.559
Yes	1.6 (1.12-2.29)		1.42 (0.67-2.081)	
Fat Invasion				
No	1 (As reference)	0.033	1 (As reference)	0.481
Yes	1.45 (1.03-2.03)		1.21 (0.711-2.07)	
Resection Margin				
Negative	1 (As reference)	0.741		
Positive	0.918 (0.553-1.52)			
Tumor Deposits				
No	1 (As reference)	0.401		
Yes	1.22 (0.765-1.95)			
Tumor Differentiation				
Moderately differentiated	1 (As reference)	<0.01	1 (As reference)	0.395
Poorly differentiated	1.61 (1.24-2.08)	<0.01	1.56 (0.898-2.045)	0.147
Well differentiated	0.319 (0.0445-2.29)	0.256	0.55 (0.073-4.169)	0.556
CA 19-9 (U/ml)				
<35	1 (As reference)	0.187		
35-200	1.03 (0.74-1.44)	0.848		
>200	1.29 (0.931-1.79)	0.125		
CA 125 (U/ml)				
<20	1 (As reference)	<0.01	1 (As reference)	0.061
>=20	1.58 (1.17-2.13)		1.56 (0.945-2.37)	
CEA (ng/ml)				
<5	1 (As reference)	0.539		
>=5	1.09 (0.821-1.46)			
CA50 (U/ml)				
<25	1 (As reference)	0.350		
>=25	1.19 (0.823-1.73)			
CA153 (U/ml)				
<25	1 (As reference)	0.053	1 (As reference)	0.125
>=25	1.9 (0.992-3.65)		1.59 (0.85-3.8)	
CA242 (U/ml)				
<29	1 (As reference)	0.130		
>=29	1.28 (0.931-1.75)			
CA724 (U/ml)				
<10	1 (As reference)	0.437		
>=10	1.2 (0.756-1.91)			
Portal Vein Resection				
No	1 (As reference)	0.708		
Yes	0.912 (0.564-1.47)			
Artery (SMA, HA, CA, LGA) Resection				
No	1 (As reference)	0.399		
Yes	1.31 (0.697-2.47)			
Adjuvant Treatment				
C+R-	1 (As reference)	0.402		
C+R+	1.13 (0.848-1.51)			

OS, overall survival; HR, hazard ratio.

### 3.3 Propensity-score matching analyses

In order to balance confounding factors which might affect survival outcome, PSM was performed by a 1:1 ratio. The potentially adjusting variables were based on the results of cox regression analysis, which included AJCC 8th N stage (p=0.04) and preoperative CA 125 level (p=0.061). A total of 93 PDAC patients with C+R- adjuvant treatment were matched with 93 patients in C+R+ group in the total cohort. The baseline characteristics between the two groups after PSM were listed in **Supplemental Table 1**. All adjusting variables were comparable after PSM. However, additional adjuvant radiotherapy still did not improve OS of PDAC patients after surgical resection and adjuvant chemotherapy (**Figure 2B**; mOS, C+R- vs C+R+, 29.2 vs 29.9 months, p=0.7).

### 3.4 Subgroup analyses

Further subgroup analyses were conducted to identify patients who may benefit from additional adjuvant radiotherapy. Disappointingly, forest plot (**Supplemental Figure 1**) did not show any survival advantage of extra radiotherapy in any of the subgroups.

**Figure 3:**
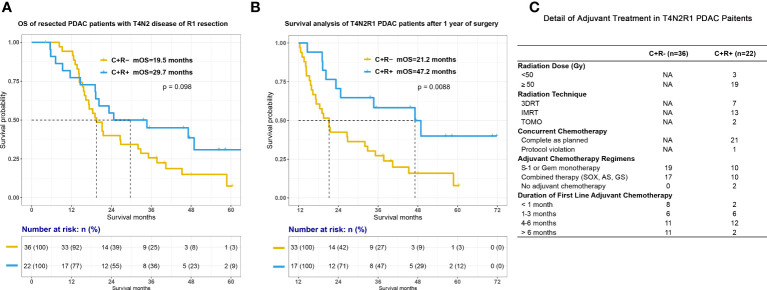
Survival between C+R- and C+R+ groups in PDAC patients with either T4, N2 disease or R1 resection. Figure showed the survival curves between two groups after surgery **(A)** and 1 year after surgical treatment **(B)**. **(C)** Detail of adjuvant treatment among T4N2R1 patients. T4N2R1, classified as pathologic T4 or N2 staging or R1 resection; Gy, gray; 3DRT, three-dimensional conformal radiation therapy; IMRT, intensity-modulated radiation therapy; TOMO, tomotherapy; Gem, gemcitabine; SOX, S-1 plus oxaliplatin; AS; nabpaclitaxel plus S-1; GS, gemcitabine plus S-1.

Based on Cox analyses, our previous prospective research and some retrospective studies, PDAC patients after curative surgery and with high-risk pathologic features may have better survival outcome with adjuvant radiotherapy (25–28, 30). We sought to examine the potential benefit of adjuvant radiotherapy among the highest-risk patients and therefore patients with either T4N2 disease or R1 resection were retrieved from the total cohort. Among this high-risk subgroup, there was a trend toward improved OS when adjuvant RT was administered (**Figure 3A**; mOS, C+R- vs C+R+, 19.5 vs 29.7 months, p=0.098). The curves diverge following the 1-year time point and landmark survival analysis for patients surviving beyond this point surprisingly demonstrated a significant difference as seen in **Figure 3B** (mOS for patients alive 1 year after surgical resection, 21.2 vs 47.2 months for the C+R- vs C+R groups, respectively, p=0.0088), although it must be noted that numbers for comparison were low (33 C+R- patients, 17 C+R+ patients).

## 4 Discussion

The role of CRT in the adjuvant setting for pancreatic cancer remains debatable due to the conflicting results from different clinical trials and retrospective studies. The reasons behind may be difference in indications of CRT and treatment protocols. While adjuvant chemotherapy has been a standard treatment for PDAC patients after curative surgery, it was critical to identify whom would benefit from additional adjuvant radiotherapy. Thus, we conducted the present retrospective study to investigate the therapeutic effect of additional adjuvant radiotherapy in PDAC patients who received surgical treatment of primary pancreatic lesion and adjuvant chemotherapy after surgery. A total of 399 patients were included, 93 of them receiving adjuvant chemoradiotherapy and 306 of them receiving adjuvant chemotherapy only. Patients in C+R+ group were more likely to be male patients with T3-4 disease. Lymph node metastases was the only negative prognostic factor associated with OS. Additional adjuvant radiotherapy did not improve OS both before and after PSM. Further subgroup analyses showed that PDAC patients with either T4N2 disease or R1 resection have increased OS with radiotherapy at 1 year after surgical treatment.

It was reasonable that no difference in OS was found between C+R- and C+R+ in total cohort. Based on CSCO and NCCN guidelines, adjuvant radiotherapy was recommended only in patients with features that portend high risk for local recurrence, for example, positive resection margin and regional lymph node metastases (6, 7). However, a high proportion of patients in this study appear to have had relatively early-stage disease (>50% stage I/>90% stage I-II in the C+R-group; 38.8% stage I/>85% stage I-II in the C+R+ group) and negative margins (93.7%). Based on our previous research and some retrospective studies, CRT showed promising efficacy in resected PDAC patients with high-risk pathologic features, including positive resection margin, pathologic T3-4 or N1-2 disease, peripancreatic fat invasion, microvascular invasion, and perineural invasion (25–28, 30). Thus, we conducted further subgroup analyses and surprisingly found that patients with T4, N2 stage or R1 resection had better OS with additional radiotherapy at 1 year after surgical resection. This result was similar with those of PREOPANC Trial, which demonstrating that neoadjuvant chemoradiotherapy shown survival advantage for resectable and borderline resectable PDAC patients after 1 year from diagnosis when compared with upfront surgery (31). As we known, biological nature should be taken into consideration when talking about resectability of pancreatic cancer. Some patients could have disease progression even after aggressive surgery and (neo)adjuvant treatments and died within 1 year after diagnosis. By conducting landmark analyses with a cutoff point at 1 year after surgical treatment, we may be able to reduce the confounding effect of these biologically unresectable PDAC patients. Besides, the underlying mechanism about poor response to radiation was nebulous. Previous study has demonstrated that genetic alterations and tumor microenvironment of individual patients could impact the response to radiation therapy. Thus, further research, which shall include the genetic and microenvironment detail of individual PDAC patients into survival analyses, is needed to reveal potential mechanism (32).

There were obviously some limitations of our study. First, this was a single institution retrospective study conducted in a high-volume pancreatic cancer center. We have specialized pancreatic disease multidisciplinary team (MDT) with experienced radiation therapists. Our research and previous studies have showed that high volume center with MDT could contribute to appropriate clinical decision making, lower mortality rate of pancreatectomies and better survival outcome (33–35). Meanwhile, we should also take into account the quality of radiotherapy which could have a significant impact on therapeutic effect of CRT, since Abrams et al. had demonstrated that failure to adherence to radiation therapy protocol was associated with poorer survival outcome in PDAC patients (29). Thus, the conclusion may not be able to generalized to other hospitals. Second, our results may need to be interpreted with attention that discrepancies in treatment protocol, especially adjuvant chemotherapy regimens, could affect survival outcome of PDAC patients after surgery. PRODIGE-24 has revealed the superiority of mFOLFIRINOX regimen compared to gemcitabine in terms of DFS and OS (8). Although we have summarized details of adjuvant treatment of T4N2R1 patients as **Figure 3C**, we were not able to collect all information about adjuvant treatment in other patients in this study. We have no idea if additional adjuvant radiotherapy could still offer survival benefit when combined adjuvant chemotherapy regimens, such as mFOLFIRINOX, nabpaclitaxel plus gemcitabine, capecitabine plus gemcitabine, were administrated in the process of adjuvant CRT. Third, we did not show the comparison of DFS in the result part. The median DFS for all included patients was around 16.4 months. We did compare DFS and the recurrence patterns between groups and the analyses showed no difference in DFS in both all included patients and T4N2R1 patients (**Supplemental Figure 3**). The reason why we did not highlight DFS is that some of the patients who received surgery between 2017 to 2019 were not able to complete their routine 3-months-follow-up in either our hospital or local medical centers on time due to the pandemic in the past 3 years in China. Although we have collected the event of disease progression through telephone follow-up, DFS was not precise enough to describe the actual survival outcome when compared with OS.

To our knowledge, further clinical trials should be undertaken in the following issues: 1) identifying potential subgroups who could benefit from adjuvant radiotherapy and also those with poor biological features and dismal prognosis even with aggressive treatment; 2) what is the best treatment combination, including chemotherapy regimens, treatment sequence, radiotherapy technique etc.

## 5 Conclusion

Additional adjuvant RT was not associated with an OS benefit across all included PDAC patients receiving surgery and adjuvant chemotherapy, though did show a possible OS benefit for the subgroup with T4N2 disease or R1 resection at 1 year after surgery.

## Data availability statement

The original contributions presented in the study are included in the article/**Supplementary Material**. Further inquiries can be directed to the corresponding authors.

## Ethics statement

The studies involving human participants were reviewed and approved by the ethical committee of our hospital (B2022-249R) and all patients have signed informed consent forms before we collected their medical records for researching purpose. The patients/participants provided their written informed consent to participate in this study.

## Author contributions

LW: project administration, data curation, investigation, methodology. YX: project administration, data curation, investigation, writing – original draft. YZ: investigation. ZZ: funding acquisition, investigation, methodology, project administration, resources. YF: investigation. DW: investigation. WW: project administration, data curation, investigation. XG: investigation. ML: software, visualization, methodology. YO: investigation. SD: funding acquisition, investigation, writing – review & editing. WL: funding acquisition, investigation, writing – review & editing. All authors contributed to the article and approved the submitted version.
