# Supplementary figures and images for: Additional adjuvant radiotherapy improves survival at 1 year after surgical treatment for pancreatic cancer patients with T4, N2 disease, positive resection margin, and receiving adjuvant chemotherapy
Source: Front Oncol. 2023 Jul 18;13:1109068. doi: 10.3389/fonc.2023.1109068 (PMC10391548; doi:10.3389/fonc.2023.1109068)

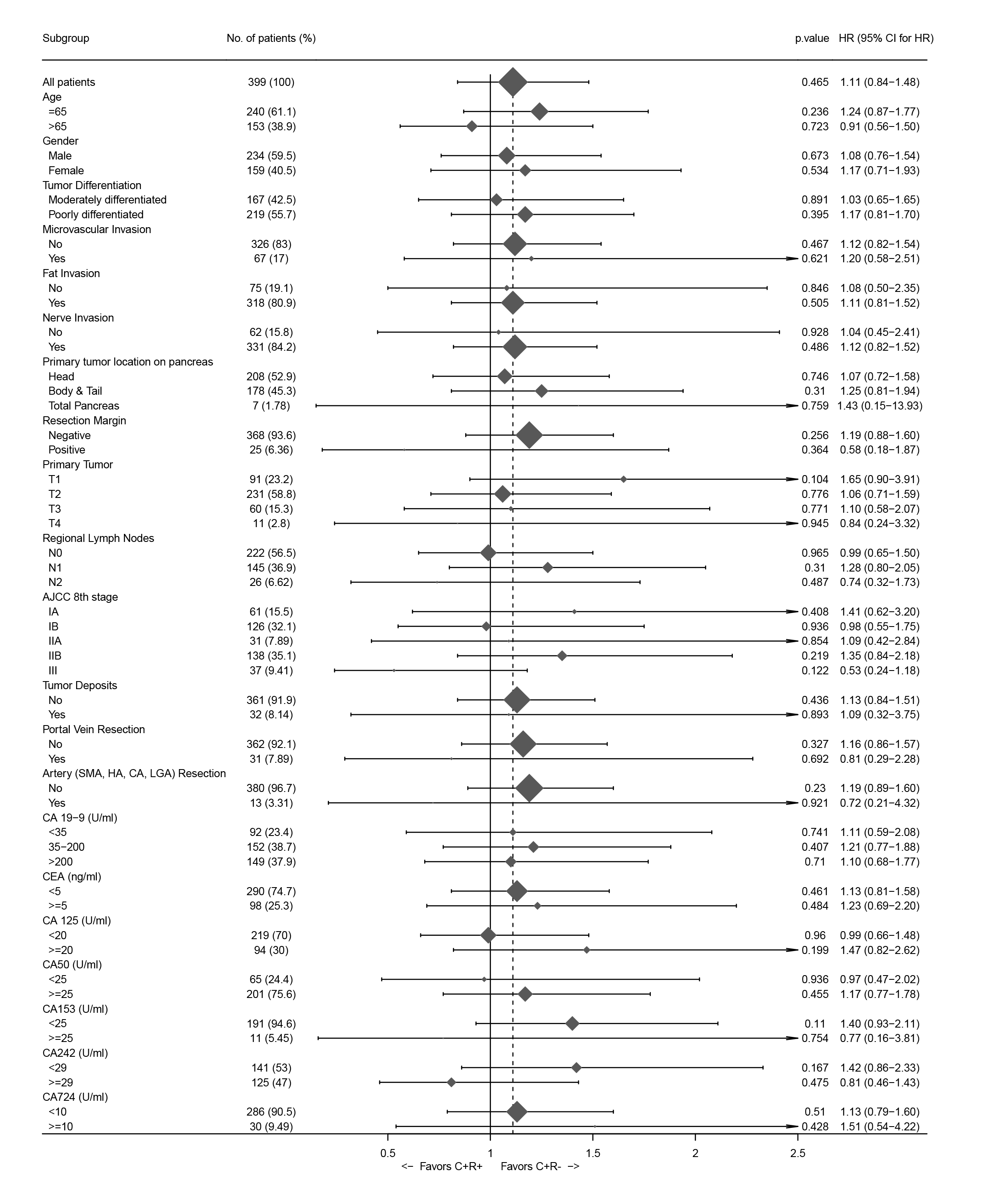

Supplement: Supplementary file 1 [file Image_1.tif]

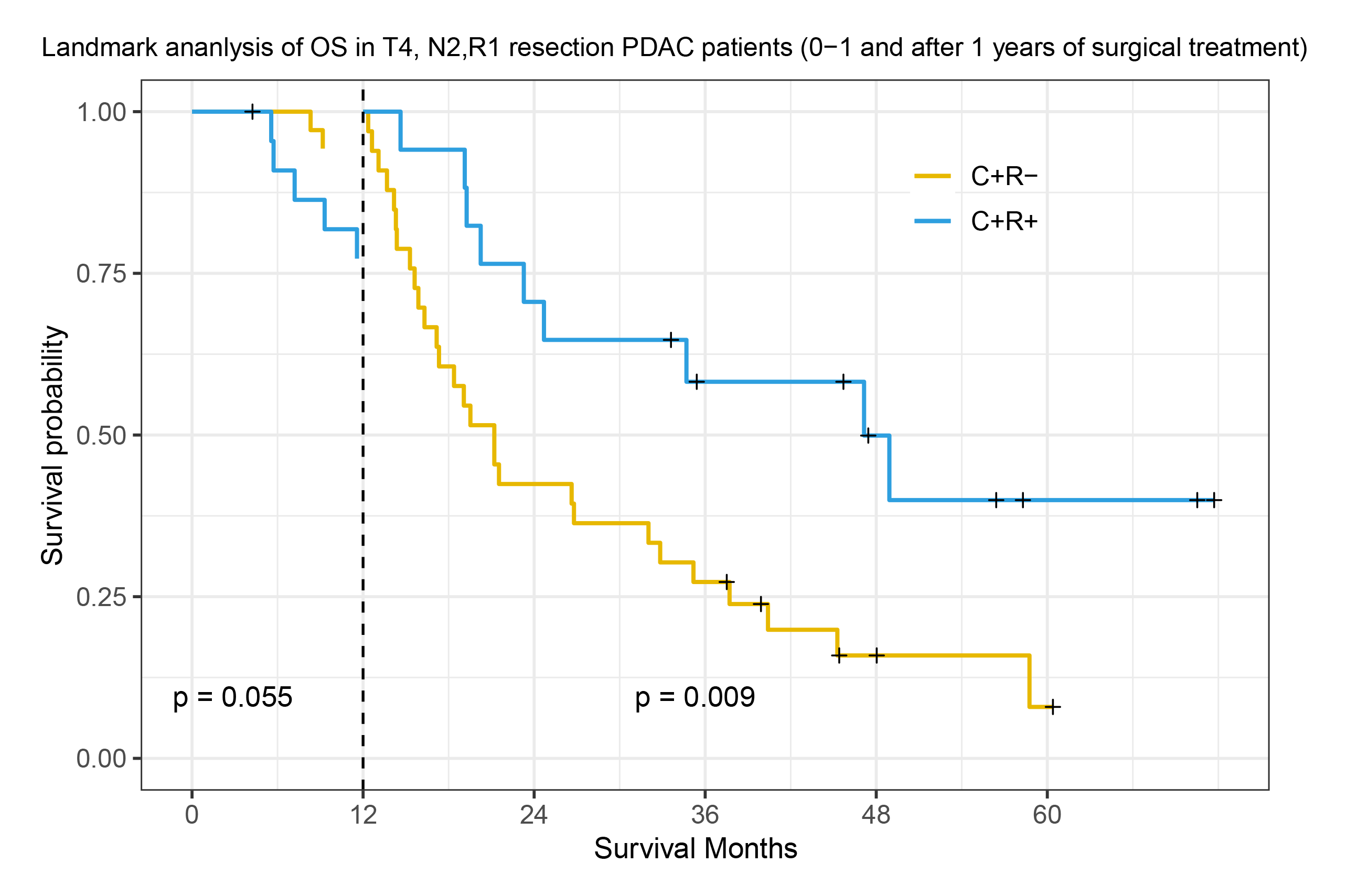

Supplement: Supplementary file 2 [file Image_2.tif]

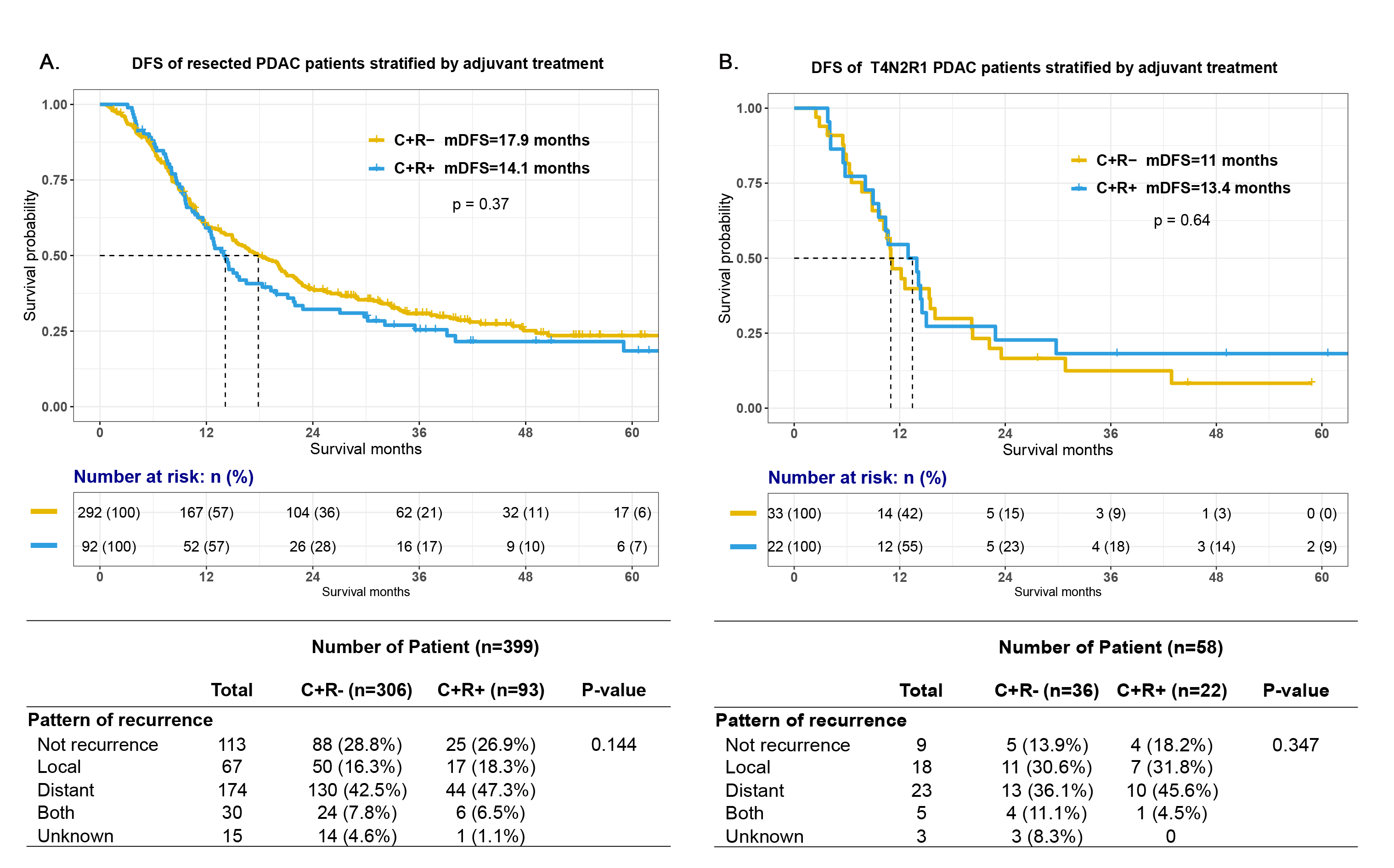

Supplement: Supplementary file 3 [file Image_3.tif]
