# Supplementary material for: Additional adjuvant radiotherapy improves survival at 1 year after surgical treatment for pancreatic cancer patients with T4, N2 disease, positive resection margin, and receiving adjuvant chemotherapy
Source: Front Oncol. 2023 Jul 18;13:1109068. doi: 10.3389/fonc.2023.1109068 (PMC10391548; doi:10.3389/fonc.2023.1109068)
Supplement: Supplementary file 4 [file Table_1.docx]

Supplemental Table 1. Baseline characteristics of patients stratified by adjuvant treatment after propensity-score matching

|  |  | **Number of Patient (n=186)** | |  |
| --- | --- | --- | --- | --- |
|  | **Total** | **C+R- (n=93)** | **C+R+ (n=93)** | **P-value** |
| **Age** | | | | |
| Mean (SD) | 62.5 (±8.0) | 63.2 (±8.2) | 61.9 (±7.8) | 0.3 |
| **Gender** | | | | |
| Male | 117 (62.9%) | 50 (53.8%) | 67 (72.0%) | 0.015 |
| Female | 69 (37.1%) | 43 (46.2%) | 26 (28.0%) |  |
| **ASA classification** | | | | |
| 1 | 31 (16.7%) | 18 (19.4%) | 13 (14.0%) | 0.81 |
| 2 | 146 (78.5%) | 70 (75.3%) | 76 (81.7%) |  |
| 3 | 7 (3.8%) | 4 (4.3%) | 3 (3.2%) |  |
| Unknown | 2 (1.1%) | 1 (1.1%) | 1 (1.1%) |  |
| **Primary tumor location on pancreas** | | | | |
| Head | 106 (57.0%) | 62 (66.7%) | 44 (47.3%) | 0.014 |
| Body & Tail | 78 (41.9%) | 30 (32.3%) | 48 (51.6%) |  |
| Total Pancreas | 2 (1.1%) | 1 (1.1%) | 1 (1.1%) |  |
| **Primary Tumor** | | | | |
| T1 | 41 (22.0%) | 17 (18.3%) | 24 (25.8%) | 0.025 |
| T2 | 105 (56.5%) | 62 (66.7%) | 43 (46.2%) |  |
| T3 | 36 (19.4%) | 12 (12.9%) | 24 (25.8%) |  |
| T4 | 4 (2.2%) | 2 (2.2%) | 2 (2.2%) |  |
| **Regional Lymph Nodes** | | | | |
| N0 | 98 (52.7%) | 49 (52.7%) | 49 (52.7%) | 1 |
| N1 | 66 (35.5%) | 33 (35.5%) | 33 (35.5%) |  |
| N2 | 22 (11.8%) | 11 (11.8%) | 11 (11.8%) |  |
| **AJCC 8th stage** | | | | |
| IA | 27 (14.5%) | 13 (14.0%) | 14 (15.1%) | 0.75 |
| IB | 51 (27.4%) | 29 (31.2%) | 22 (23.7%) |  |
| IIA | 18 (9.7%) | 7 (7.5%) | 11 (11.8%) |  |
| IIB | 64 (34.4%) | 31 (33.3%) | 33 (35.5%) |  |
| III | 26 (14.0%) | 13 (14.0%) | 13 (14.0%) |  |
| **Microvascular Invasion** | | | | |
| No | 159 (85.5%) | 79 (84.9%) | 80 (86.0%) | 1 |
| Yes | 27 (14.5%) | 14 (15.1%) | 13 (14.0%) |  |
| **Nerve Invasion** | | | | |
| No | 28 (15.1%) | 16 (17.2%) | 12 (12.9%) | 0.54 |
| Yes | 158 (84.9%) | 77 (82.8%) | 81 (87.1%) |  |
| **Fat Invasion** | | | | |
| No | 29 (15.6%) | 13 (14.0%) | 16 (17.2%) | 0.69 |
| Yes | 157 (84.4%) | 80 (86.0%) | 77 (82.8%) |  |
| **Resection Margin** | | | | |
| Negative | 174 (93.5%) | 90 (96.8%) | 84 (90.3%) | 0.13 |
| Positive | 12 (6.5%) | 3 (3.2%) | 9 (9.7%) |  |
| **Tumor Deposits** | | | | |
| No | 173 (93.0%) | 85 (91.4%) | 88 (94.6%) | 0.57 |
| Yes | 13 (7.0%) | 8 (8.6%) | 5 (5.4%) |  |
| **Tumor Differentiation** | | | | |
| Well differentiated | 1 (0.5%) | 1 (1.1%) | 0 (0.0%) | 0.072 |
| Moderately differentiated | 70 (37.6%) | 28 (30.1%) | 42 (45.2%) |  |
| Poorly differentiated | 113 (60.8%) | 63 (67.7%) | 50 (53.8%) |  |
| Unknown | 2 (1.1%) | 1 (1.1%) | 1 (1.1%) |  |
| **CA 19-9 (U/ml)** | | | | |
| <35 | 43 (23.1%) | 23 (24.7%) | 20 (21.5%) | 0.4 |
| 35-200 | 77 (41.4%) | 34 (36.6%) | 43 (46.2%) |  |
| >200 | 66 (35.5%) | 36 (38.7%) | 30 (32.3%) |  |
| **CA 125 (U/ml)** | | | | |
| <20 | 103 (72.0%) | 50 (71.4%) | 53 (72.6%) | 1 |
| >=20 | 40 (28.0%) | 20 (28.6%) | 20 (27.4%) |  |
| **CEA (ng/ml)** | | | | |
| <5 | 142 (76.8%) | 73 (78.5%) | 69 (75.0%) | 0.61 |
| >=5 | 43 (23.2%) | 20 (21.5%) | 23 (25.0%) |  |
| **CA50 (U/ml)** | | | | |
| <25 | 29 (25.0%) | 12 (22.2%) | 17 (27.4%) | 0.67 |
| >=25 | 87 (75.0%) | 42 (77.8%) | 45 (72.6%) |  |
| **CA153 (U/ml)** | | | | |
| <25 | 73 (94.8%) | 21 (95.5%) | 52 (94.5%) | 1 |
| >=25 | 4 (5.2%) | 1 (4.5%) | 3 (5.5%) |  |
| **CA242 (U/ml)** | | | | |
| <29 | 59 (53.6%) | 25 (52.1%) | 34 (54.8%) | 0.85 |
| >=29 | 51 (46.4%) | 23 (47.9%) | 28 (45.2%) |  |
| **CA724 (U/ml)** | | | | |
| <10 | 131 (90.3%) | 63 (87.5%) | 68 (93.2%) | 0.28 |
| >=10 | 14 (9.7%) | 9 (12.5%) | 5 (6.8%) |  |
| **Portal Vein Resection** | | | | |
| No | 171 (91.9%) | 87 (93.5%) | 84 (90.3%) | 0.59 |
| Yes | 15 (8.1%) | 6 (6.5%) | 9 (9.7%) |  |
| **Artery (SMA, HA, CA, LGA) Resection** | | | | |
| No | 182 (97.8%) | 91 (97.8%) | 91 (97.8%) | 1 |
| Yes | 4 (2.2%) | 2 (2.2%) | 2 (2.2%) |  |

SD, standard deviation; ASA, American Society of Anesthesiologists; AJCC, American Joint Committee on Cancer; CA 19-9, carbohydrate antigen 19-9; CA 125, carbohydrate antigen 125; CEA, carcinoembryonic antigen; AFP, alpha-fetoprotein; CA 50, carbohydrate antigen 50; CA 153, carbohydrate antigen 50; CA 242, carbohydrate antigen 242; CA 724, carbohydrate antigen 724; SMA, superior mesenteric artery; HA, hepatic artery; CA, celiac trunk; LGA, left gastric artery.
